# Cord Blood Bilirubin, Albumin, and Hemoglobin as Predictors of Significant Hyperbilirubinemia in ABO-Incompatible Newborns: A Prospective Study in a Tertiary Neonatal Center in India

**DOI:** 10.7759/cureus.94975

**Published:** 2025-10-20

**Authors:** Jefferson George, Indhu Poomalai, Dorian H Terrence, Lavanya Murugesh

**Affiliations:** 1 Trauma and Orthopedic Surgery, University Hospitals of Leicester NHS Trust, Leicester, GBR; 2 Medicine and Surgery, Madurai Medical College and Government Rajaji Hospital, Madurai, IND; 3 Surgical Oncology, Government Arignar Anna Memorial Cancer Hospital and Research Institute, Kanchipuram, IND; 4 Pediatrics and Child Health, Government Theni Medical College and Hospital, Theni, IND

**Keywords:** abo incompatibility, hyperbilirubinemia, neonatal jaundice, phototherapy, resource-limited settings

## Abstract

Background and aim

ABO incompatibility is a common cause of neonatal hyperbilirubinemia. Early identification of babies at risk of neonatal jaundice allows timely treatment and helps minimize the risk of kernicterus. Given the paucity of data on Indian neonates with ABO incompatibility, this study aimed to identify reliable cord blood predictors of early hyperbilirubinemia by evaluating umbilical cord bilirubin, albumin, and hemoglobin levels in ABO-incompatible newborns.

Methods

This study was conducted at a tertiary care hospital in India and included 210 babies with blood groups A+ve, B+ve, or AB+ve born to mothers with blood group O+ve. All included neonates had a gestational age >37 weeks and a birth weight between 2.5 and 4 kg. Cord blood bilirubin, albumin, hemoglobin, and blood grouping and typing were evaluated at birth. Newborns were monitored for jaundice until the fifth postnatal day and further assessed by measuring total, direct, and indirect bilirubin levels.

Results

Of the 210 babies studied, 156 (74.28%) developed jaundice requiring phototherapy, 19 (9%) developed jaundice but did not require phototherapy, and 35 (16.7%) did not develop jaundice. The mean cord bilirubin level (2.8 mg/dl) was higher, while mean cord albumin (2.64 g/dl) and hemoglobin (14.41 g/dl) levels were lower among newborns who developed jaundice requiring phototherapy compared with those who did not. Babies with cord bilirubin >2.3 mg/dl, cord hemoglobin <15 g/dl, and cord albumin ≤2.5 g/dl were more likely to develop significant hyperbilirubinemia associated with ABO incompatibility.

Conclusions

Umbilical cord blood levels of bilirubin, albumin, and hemoglobin demonstrated potential as early predictors of significant hyperbilirubinemia in ABO-incompatible term neonates. Incorporating cord blood screening into routine neonatal care could facilitate early identification and timely intervention, particularly in resource-limited settings. However, further validation is needed before clinical implementation.

## Introduction

Neonatal hyperbilirubinemia is a common cause for concern, affecting nearly 60% of term babies within the first seven days after birth [1,2]. It is also the most frequent cause of readmission during the early neonatal period. While most cases are physiological, a small subset may progress to severe hyperbilirubinemia, posing irreversible complications such as acute bilirubin encephalopathy and kernicterus [3]. ABO incompatibility occurs in about 15-25% of pregnancies [4], and only 1% of these babies develop significant hyperbilirubinemia requiring treatment [5].

ABO incompatibility continues to be a common cause of neonatal jaundice worldwide. It occurs when babies with blood groups A, B, or AB are born to mothers with blood group O. ABO hemolytic disease is relatively mild due to the low antigenicity of A and B factors and their widespread expression in tissues other than red blood cells [6]. However, a small proportion of these infants develop early-onset hyperbilirubinemia requiring prompt recognition and management.

Early identification of newborns at risk of significant jaundice remains a challenge [7]. Early-onset jaundice, typically within the first 24-72 hours of life, is the initial sign of hyperbilirubinemia due to ABO incompatibility. Other signs, such as anemia, mild splenomegaly, and hydrops fetalis, are rare [8]. A positive Coombs test may be observed in babies with moderate to severe hyperbilirubinemia; however, the reaction is often weak, with a high rate of false negatives [9]. Moreover, early postpartum discharge of healthy term newborns has become a common practice in low- and middle-income countries. In such settings, where follow-up may be limited, recognizing and treating jaundice early has become increasingly difficult [10].

The American Academy of Pediatrics (AAP) recommends that newborns discharged within 48 hours should have a follow-up visit 48-72 hours after discharge to assess for significant jaundice and other issues [11]. However, adherence to this guideline is challenging in resource-limited settings, where follow-up infrastructure and accessibility may be restricted. This scenario highlights the need for an early, noninvasive, and reliable screening tool for risk identification.

Previous studies have evaluated the predictive value of cord blood bilirubin, albumin, and hemoglobin in identifying significant hyperbilirubinemia [12-16]. However, few studies have examined these markers specifically in ABO-incompatible newborns within the Indian healthcare context.

This study was conducted to evaluate the predictive value of cord bilirubin, albumin, and hemoglobin levels in identifying ABO-incompatible term neonates at risk of developing significant hyperbilirubinemia in the Indian setting. The primary objective was to determine predictive thresholds for each of these cord blood parameters in identifying neonates at risk of developing clinically significant hyperbilirubinemia. The secondary objective was to assess the correlation between cord bilirubin and serum bilirubin levels measured at 72 hours of life. By identifying early and reliable biomarkers at birth, this study aims to contribute to the development of risk-based screening strategies, particularly in low-resource settings where post-discharge follow-up may be limited, and to inform clinical decision-making regarding interventions such as phototherapy.

## Materials and methods

Study design and setting

A prospective study was conducted in the Department of Pediatrics at Government Theni Medical College and Hospital, India, a tertiary care center, over a period of one year (March 2022 to March 2023).

Study participants

Healthy term neonates with ABO incompatibility who met the inclusion and exclusion criteria were included in the study (Table 1).

**Table 1:** Inclusion and exclusion criteria

Inclusion criteria	Exclusion criteria
Term (>37 weeks)	Preterm
Birth weight: 2.5-4 kg	Intrauterine growth restriction/small for gestational age
APGAR score ≥7	Asphyxiated babies
Normal vaginal delivery or lower segment cesarean section	Instrumental delivery
Newborns born to ‘O’ positive mothers with baby blood group A, B, or AB at GTMCH, Theni	Rh incompatibility
	Infant of a diabetic mother
	At risk for sepsis
	Associated congenital anomalies

Data collection

After enrollment and obtaining informed consent from the parents, blood samples were collected from the umbilical cords of babies meeting the inclusion criteria. These neonates were monitored daily using the Kramer scale to identify the development of jaundice. All babies were further evaluated with serum total, direct, and indirect bilirubin measurements at 72 hours of life.

The monitoring period was aligned with the clinical course of ABO incompatibility, which predominantly causes early-onset hemolysis and peak bilirubin levels within the first three to five days, while also accommodating institutional discharge protocols. Babies who developed jaundice requiring phototherapy or exchange transfusion, as per the AAP guidelines, were considered to have significant hyperbilirubinemia, which was taken as the primary outcome of the study.

Significant hyperbilirubinemia was defined according to the 2004 AAP Subcommittee on Hyperbilirubinemia guidelines [11]. Specifically, any neonate whose total serum bilirubin (TSB) level met or exceeded the age-specific threshold for phototherapy as outlined in the AAP phototherapy nomogram (for infants ≥35 weeks’ gestation) was classified as having significant hyperbilirubinemia. The thresholds on the nomogram are based on the infant’s postnatal age in hours, gestational age, and the presence of risk factors.

Serum bilirubin was estimated from peripheral venous samples using the Diazo method, a well-established and commonly employed colorimetric assay in clinical biochemistry [17]. Babies diagnosed with significant hyperbilirubinemia were followed up until discharge, according to hospital protocol.

The direct antiglobulin test (DAT) was not performed systematically in our cohort due to institutional protocol limitations and cost considerations. Although DAT is used to detect antibody-mediated hemolysis, it has limited sensitivity in cases of ABO incompatibility [18] and was therefore excluded from this study.

The sample size was calculated based on a previous study reporting a sensitivity of 82.5% for cord bilirubin levels ≥1.79 mg/dl in predicting significant neonatal hyperbilirubinemia [19]. For this study, a target sensitivity of 75%, a 95% confidence level, and a 60% prevalence of significant hyperbilirubinemia among term neonates in our setting were assumed. The minimum required total sample size was estimated to be 122. To account for potential dropouts, incomplete follow-up, or missing data, an additional 30% was added, resulting in a revised estimate of 159 participants.

Participants were enrolled through a consecutive sampling approach, whereby all eligible neonates presenting during the study period were prospectively recruited. However, due to practical constraints such as consent availability and resource limitations, an element of convenience sampling was introduced. Efforts were made to ensure continuous recruitment throughout the study duration to minimize temporal bias. Ultimately, 210 neonates were enrolled to ensure sufficient statistical power and data completeness.

Statistical analysis

Continuous variables were analyzed descriptively using mean and SD, while categorical data were expressed as numbers and percentages. One-way ANOVA was used to compare the means of the analytical variables among the three groups. Pearson correlation coefficients were calculated to assess the strength and direction of linear relationships between variables.

A binary logistic regression model was fitted with significant hyperbilirubinemia (yes/no) as the dependent variable and cord hemoglobin, albumin, and bilirubin levels as independent variables. The specific cutoff values of each parameter most predictive of significant hyperbilirubinemia were determined using receiver operating characteristic (ROC) curve analysis, and their sensitivity and specificity were estimated. A p-value <0.05 was considered statistically significant.

Data were analyzed using IBM SPSS Statistics for Windows, Version 28.0 (Released 2021; IBM Corp., Armonk, NY, USA) and Microsoft Excel (Microsoft Corporation, Redmond, WA, USA).

Ethical considerations

Ethical approval for the study was obtained from the Institutional Ethics Committee of Government Theni Medical College, Theni, India (approval 1515/MEIII/21, dated February 28, 2022). Written informed consent was obtained from the mothers of all neonates enrolled in the study.

## Results

A total of 210 neonates were included in this study. Of these, 156 (74.28%) developed jaundice requiring phototherapy and/or exchange transfusion (significant hyperbilirubinemia), 19 (~9%) developed jaundice but did not require phototherapy (nonsignificant hyperbilirubinemia), and 35 (16.7%) did not develop jaundice. The baseline characteristics of the study population are summarized in Table 2.

**Table 2:** Summary of baseline characteristics ^*^ The chi-square test of independence was used for gender and mode of delivery. ^**^ ANOVA was used for all other variables. Birth weight, cord hemoglobin, cord albumin, cord bilirubin, and TSB are expressed as mean ± SD. Gender and mode of delivery are expressed as numbers and percentages. TSB, total serum bilirubin

Variable	No jaundice 35 (16.5%)	Nonsignificant hyperbilirubinemia 19 (9%)	Significant hyperbilirubinemia 156 (74.28%)	Test statistic value	p-Value
Birth weight (kg)	3.03 ± 0.38	3.04 ± 0.42	3.10 ± 0.37	0.57	0.564^**^
Male	13 (6%)	9 (4.2%)	76 (36.2%)	3.91	0.418^*^
Female	22 (10.5%)	10 (4.7%)	80 (38.1%)	-	-
Cesarean delivery	20 (9.5%)	14 (6.7%)	100 (47.6%)	1.48	0.477^*^
Normal delivery	15 (7.14%)	5 (2.3%)	56 (26.6%)	-	-
Cord hemoglobin (g/dL)	15.57 ± 0.21	14.71 ± 0.48	14.41 ± 0.37	148.69	<0.001^**^
Cord albumin (g/dL)	3.14 ± 0.12	2.78 ± 0.48	2.64 ± 0.31	38.08	<0.001^**^
Cord bilirubin (mg/dL)	1.90 ± 0.14	2.52 ± 0.37	2.80 ± 0.31	134.26	<0.001^**^
TSB at 72 hours (mg/dL)	13.69 ± 0.51	15.09 ± 1.35	16.82 ± 1.22	115.21	<0.001^**^

Based on the Pearson correlation matrix (Table 3), cord bilirubin was positively correlated with TSB at 72 hours (r = 0.62). Cord hemoglobin and cord albumin were negatively correlated with TSB at 72 hours (r = -0.69 and r = -0.51, respectively).

**Table 3:** Correlation matrix of cord blood biomarkers and serum bilirubin at 72 hours Pearson correlation coefficients among four neonatal biomarkers, such as cord hemoglobin, cord albumin, cord bilirubin, and TSB at 72 hours, are presented. Values range from -1 to +1, indicating the direction and strength of the linear associations between pairs of variables. TSB, total serum bilirubin

Variable	Cord hemoglobin	Cord albumin	Cord bilirubin	TSB at 72 hours
Cord hemoglobin	1	0.55	-0.65	-0.69
Cord albumin	0.55	1	-0.57	-0.51
Cord bilirubin	-0.65	-0.57	1	0.62
TSB at 72 hours	-0.69	-0.51	0.62	1

A logistic regression model (Table 4) was fitted to predict significant hyperbilirubinemia using cord hemoglobin, albumin, and bilirubin levels.

**Table 4:** Logistic regression analysis for predicting significant hyperbilirubinemia Logistic regression model examining the relationship between neonatal cord blood biomarkers (hemoglobin, albumin, and bilirubin) and the likelihood of developing significant hyperbilirubinemia.

Parameter	Coefficient	Standard error	z-Value	p-Value	95% CI for coefficient
Intercept	31.47	12.95	2.43	0.015	(6.09, 56.84)
Cord hemoglobin	-1.88	0.79	-2.37	0.018	(-3.43, -0.33)
Cord albumin	-3.41	1.26	-2.70	0.007	(-5.88, -0.94)
Cord bilirubin	2.7	0.97	2.8	0.005	(0.81, 4.60)

The logistic regression analysis (Table 4) demonstrated that all three cord blood parameters, such as hemoglobin, albumin, and bilirubin, were statistically significant predictors of significant hyperbilirubinemia. Cord hemoglobin showed a significant negative association with the outcome (β = -1.88, p = 0.018), indicating that higher hemoglobin levels were associated with a lower risk of developing significant hyperbilirubinemia. Similarly, cord albumin also exhibited a significant inverse relationship (β = -3.41, p = 0.007), suggesting a potential protective role. In contrast, cord bilirubin was positively associated with the likelihood of significant hyperbilirubinemia (β = 2.70, p = 0.005), highlighting its potential utility as an early predictive biomarker.

Notably, the 95% CIs for all three predictors did not cross zero, further supporting the statistical robustness and reliability of these associations. The analysis also indicated the absence of significant multicollinearity, as all predictors had variance inflation factor values <2 (bilirubin: 1.45, hemoglobin: 1.68, albumin: 1.37), confirming the stability of the regression estimates.

**Figure 1:**
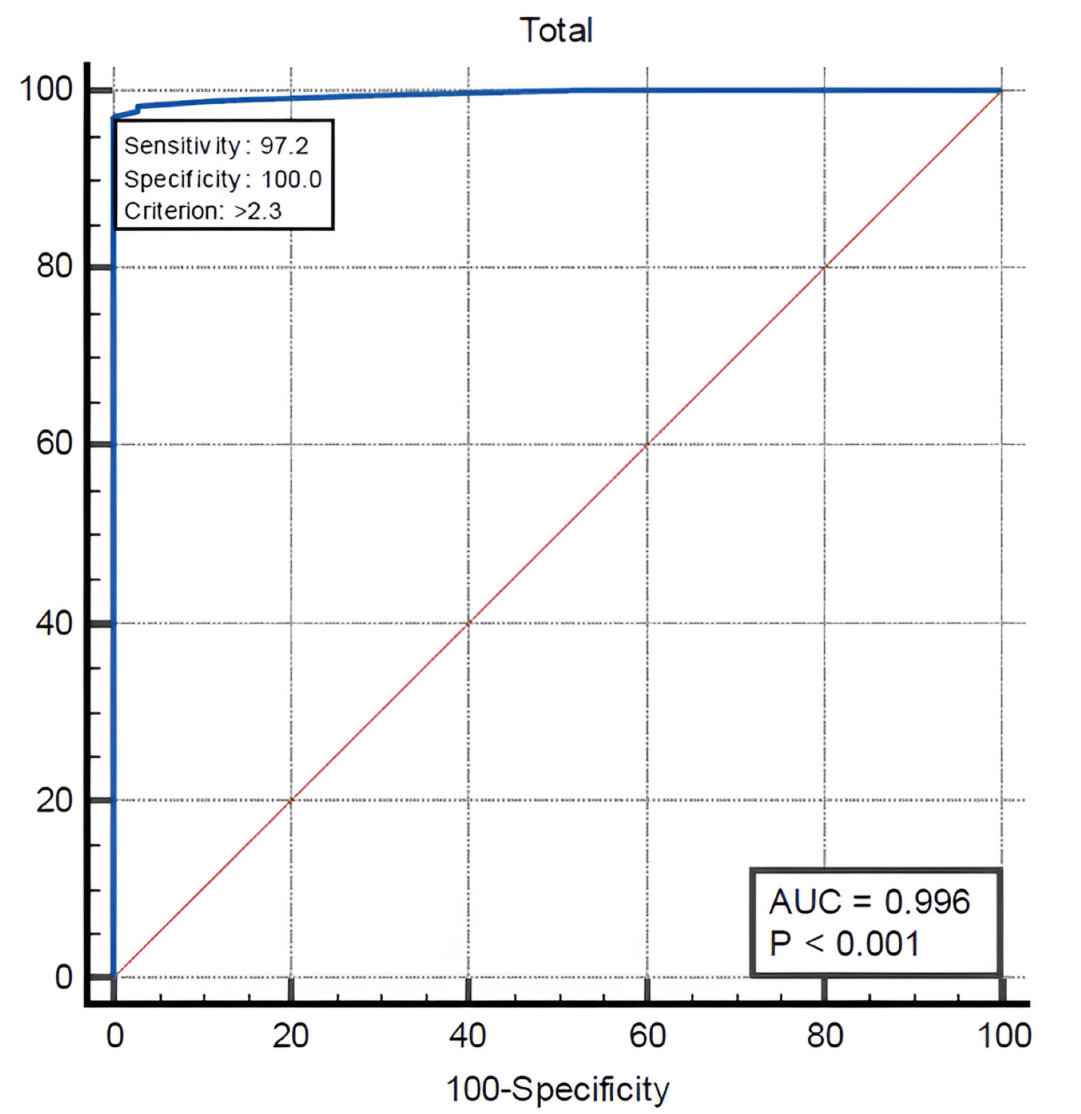
ROC curve for cord blood bilirubin AUC, area under the curve; ROC, receiver operating characteristic

ROC analysis revealed that cord bilirubin had strong predictive utility for identifying neonates at risk of significant hyperbilirubinemia. The area under the curve (AUC) was 0.996 (95% CI: 0.975-1.000, p < 0.0001). The optimal threshold was determined to be >2.3 mg/dL, yielding a sensitivity of 97.16% and specificity of 100%, with a Youden Index of 0.9716 (Figure 1).

**Figure 2:**
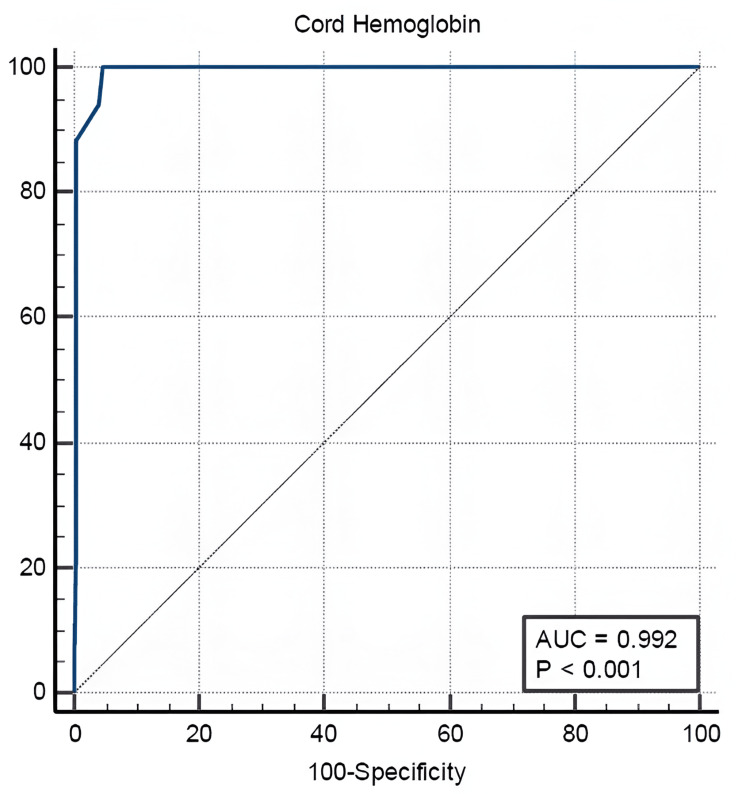
ROC curve for cord blood hemoglobin AUC, area under the curve; ROC, receiver operating characteristic

ROC analysis also demonstrated that cord blood hemoglobin predicted neonatal hyperbilirubinemia. The AUC was 0.992 (95% CI: 0.968-0.999, p = 0.0001). The optimal threshold was determined to be <15 g/dL, with a sensitivity of 100% and specificity of 95.45%, corresponding to a Youden Index of 0.9545 (Figure 2).

**Figure 3:**
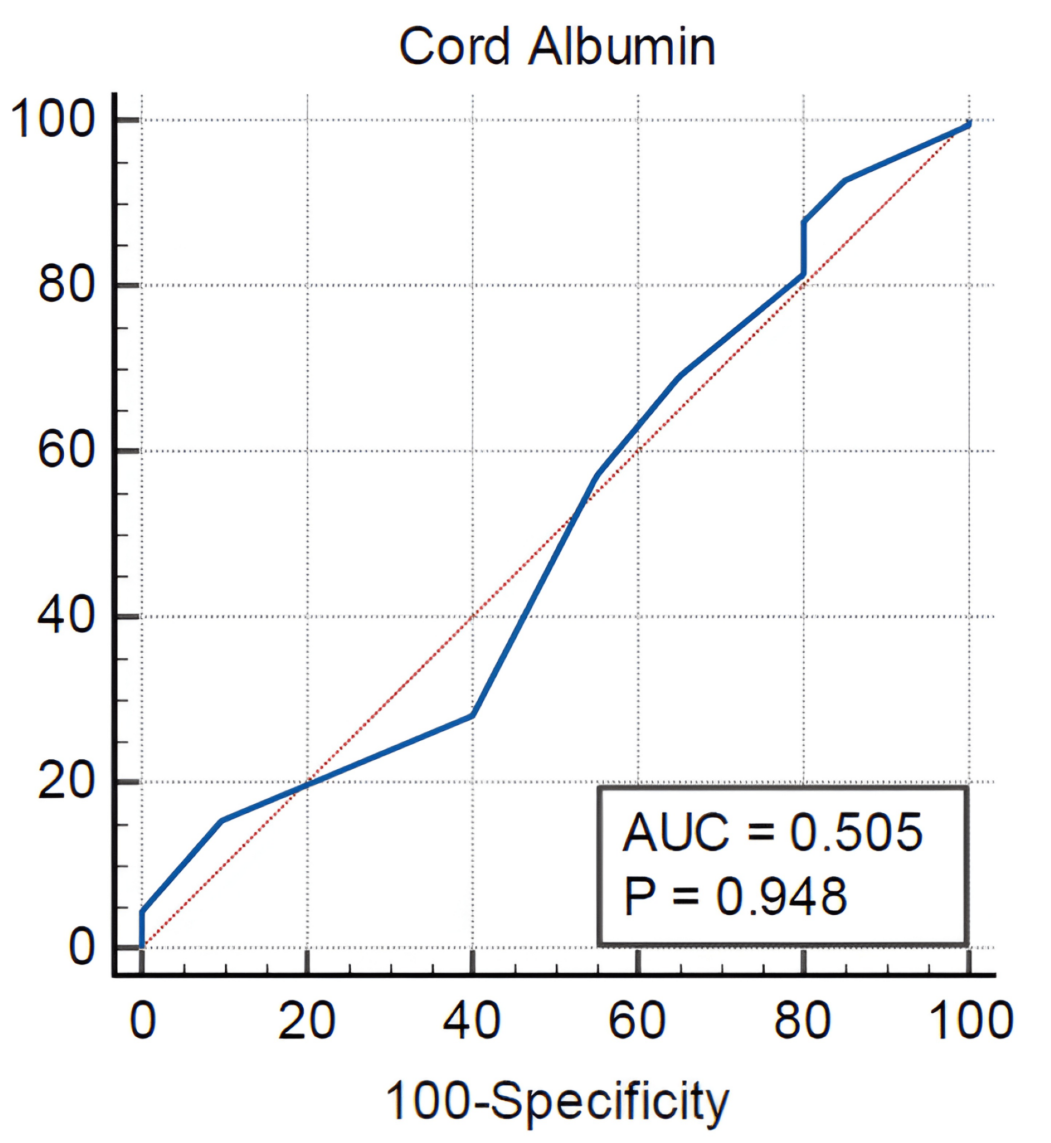
ROC curve for cord blood albumin AUC, area under the curve; ROC, receiver operating characteristic

For albumin, the AUC was 0.505 (95% CI: 0.429-0.581, p = 0.9477), indicating limited predictive utility. The identified cutoff value was ≤2.5 g/dL, which yielded a sensitivity of 28.21% and specificity of 60%, with a Youden Index of 0.1179 (Figure 3).

ROC analysis demonstrated that a cord bilirubin level greater than 2.3 mg/dL predicted the occurrence of significant hyperbilirubinemia in ABO-incompatible newborns, with a sensitivity of 97.16% and specificity of 100%. A cord blood albumin level of ≤2.5 g/dL showed a sensitivity of 28.21% and specificity of 60% for predicting significant hyperbilirubinemia in this population. In contrast, a cord hemoglobin level below 15 g/dL exhibited 100% sensitivity and 95.45% specificity, indicating it is a significant marker for identifying newborns at risk.

Among the 156 babies who developed significant jaundice, one required exchange transfusion and had cord bilirubin, hemoglobin, and albumin values of 3.8 mg/dL, 13.8 g/dL, and 2.4 g/dL, respectively. For cord bilirubin >2.3 mg/dL, the positive predictive value (PPV) was 100% and the negative predictive value (NPV) was 92.4%, indicating strong discriminatory power in identifying neonates at risk for significant hyperbilirubinemia. Similarly, cord hemoglobin <15 g/dL yielded a PPV of 98.4% and an NPV of 100%. In contrast, cord albumin ≤2.5 g/dL demonstrated lower predictive performance, with a PPV of 67.1% and a notably low NPV of 22.4%.

## Discussion

Neonatal jaundice affects nearly 60% of term and 80% of preterm newborns in the first week of life [20]. It is important to differentiate between physiological and pathological jaundice due to the risk of neurological sequelae, which can be devastating. ABO incompatibility is a significant cause of neonatal jaundice that can be managed effectively if detected early [21]. This study identified a simple test to predict significant hyperbilirubinemia in neonates with ABO incompatibility.

A substantial proportion of ABO-incompatible neonates developed significant hyperbilirubinemia (n = 156) in this study. Only 16.7% of babies remained jaundice-free. A high burden of hyperbilirubinemia among newborns with ABO incompatibility, with progression to levels requiring medical intervention, was observed. Similar trends have been reported in previous studies, highlighting the clinical importance of early risk identification and proactive monitoring of bilirubin levels in this population [21-23].

A key finding of this study was that a cord bilirubin level >2.3 mg/dL was an effective early predictor of significant hyperbilirubinemia in ABO-incompatible newborns. This threshold demonstrated a sensitivity of 97.16% and a specificity of 100%, making it a robust early screening tool given the high number of babies who developed significant hyperbilirubinemia due to ABO incompatibility. Previous studies support this approach: Singh and Jain [19] reported a lower threshold of ≥1.79 mg/dL with a sensitivity of 82.5% and a specificity of 55%, Krishnan et al. [24] reported a cutoff of >1.8 mg/dL with a sensitivity of 72% and a specificity of 80%, and Sun et al. [15] suggested a threshold of ≥2 mg/dL with a sensitivity of 68.27%. Given its relatively noninvasive nature and practical applicability, cord bilirubin testing at birth can serve as a useful screening tool, particularly in resource-limited settings with inconsistent follow-up.

Lower cord albumin levels (≤2.5 g/dL) were significantly associated with the development of hyperbilirubinemia. Albumin buffers unconjugated bilirubin, limiting its free circulating form [25]. Reduced albumin impairs this buffering capacity, contributing to elevated bilirubin levels. Sharma et al. [26] reported a similar albumin value of <3.1 g/dL with a sensitivity of 40.8% and a specificity of 34.8% in predicting significant hyperbilirubinemia, while Khairy et al. [27] found that most newborns with significant hyperbilirubinemia had low cord serum albumin (<2.8 mg/dL). Based on these physiological mechanisms and previous findings, cord albumin and hemoglobin provide valuable complementary diagnostic information for newborns at risk of clinically significant jaundice.

A cord hemoglobin cutoff of <15 g/dL demonstrated 100% sensitivity and 95.45% specificity for predicting significant hyperbilirubinemia in this study. Reduced hemoglobin levels may reflect hemolysis in ABO incompatibility. This finding aligns with Krishnan et al. [24], who reported <15.1 g/dL as associated with higher risk of significant hyperbilirubinemia in ABO-incompatible neonates. Considering the sensitivity and specificity of cord bilirubin, albumin, and hemoglobin, an effective early screening tool can be developed to guide observation strategies, early intervention, and parental education.

While no significant association was found between sex, mode of delivery, or birth weight and hyperbilirubinemia, a notable observation was that 75% of newborns who developed significant hyperbilirubinemia had a B-positive blood group. Some studies suggest that B-group babies have higher rates of direct Coombs positivity and require more interventions such as phototherapy, IVIG, or transfusion compared with group A babies [28]. However, this finding is not consistent across all studies [29].

The strengths of this study include its prospective design, which minimized recall bias, and the use of well-defined inclusion criteria and objective measurements, ensuring standardized and reproducible data. The large sample size enhanced the robustness of analyses and provided valuable insight into a potentially under-monitored condition in the Indian pediatric context.

Limitations include its single-center design and follow-up limited to the fifth postnatal day; any jaundice developing beyond this period was not captured due to study design and institutional constraints. The study did not control for all possible confounding variables, such as G6PD deficiency, sibling history of jaundice, maternal parity, or detailed feeding patterns, including exclusive breastfeeding duration and weight loss trajectories. These factors may contribute to hyperbilirubinemia and should be considered in future studies with broader data collection frameworks.

Although this study demonstrates the utility of cord blood tests as screening tools, a formal cost-effectiveness analysis was not performed to evaluate feasibility in different public healthcare systems. While consecutive sampling was employed, practical constraints, including timing of consent, parental baseline educational levels, resource availability, and workflow limitations, introduced elements of convenience sampling. Although systematic inclusion aimed to minimize selection bias, this hybrid approach may impact external validity. Future studies using randomized recruitment strategies and clinical impact modeling are recommended to validate these results in broader neonatal populations.

DAT positivity was considered but not used as an inclusion criterion due to its limited sensitivity in ABO incompatibility, especially in clinically significant but DAT-negative cases. ABO incompatibility typically exhibits low antigen density, limiting DAT’s diagnostic value. Our approach, which included all ABO-incompatible neonates regardless of DAT status, enhances clinical applicability and reflects real-world diagnostic limitations. Nevertheless, future studies integrating DAT and additional hemolytic indices may further stratify pathophysiological risk.

## Conclusions

The findings of this study provide strong evidence for the potential use of cord blood parameters, namely, bilirubin, albumin, and hemoglobin, as early indicators of significant hyperbilirubinemia in ABO-incompatible newborns. Within the limitations of this study, these findings should be considered preliminary predictive associations. Formal calibration or internal validation methods required for clinical prediction models were not performed. Nonetheless, these exploratory results can form the basis for future research into early risk calculation and predictive accuracy, with further studies needed to assess model calibration, stability, and clinical impact in larger and more diverse populations. This is particularly relevant in low- and middle-income healthcare settings, where early postpartum discharge is common and follow-up or safety-netting mechanisms may be inconsistent. Delayed detection of hyperbilirubinemia can be dangerous due to the risk of neurotoxicity and kernicterus. Incorporating cord blood tests as a screening tool may also facilitate parental counselling and education on follow-up care. Observational studies such as ours can inform larger multicenter studies that include broader populations, control for confounders, and incorporate cost-effectiveness analyses. Future research should focus on diverse populations, ethnic groups, and different healthcare settings to assess clinical utility, including potential reductions in intervention requirements and overall costs. Confounders such as G6PD status should be considered to develop a comprehensive risk assessment model. Implementing routine cord blood testing posed practical challenges in this study, including delayed laboratory turnaround, cost constraints, staff training requirements, and cultural resistance. These challenges highlight potential barriers to implementation and underscore the need for standardized protocols to ensure feasibility across varied clinical settings.

Cord blood analysis at birth is a reliable and noninvasive method for early risk stratification. Our results contribute to the growing evidence supporting early identification strategies for neonatal hyperbilirubinemia, particularly in resource-constrained settings. Routine implementation could help guide timely phototherapy and other relevant treatments in situations where delayed diagnosis is a concern. However, broader adoption requires careful resource assessment, cost-effectiveness evaluation, and protocol adjustments at each institution to ensure sustainability. With early screening, healthcare providers can proactively manage the incidence of significant hyperbilirubinemia and reduce the risk of associated complications.
